# Emergency guidelines and emergency cards

**DOI:** 10.1186/1750-1172-9-S1-O15

**Published:** 2014-11-11

**Authors:** Véronique Faucounneau, Ana Rath

**Affiliations:** 1 INSERM, US14 – Orphanet, Paris, 75014, France

## 

Rare diseases are not well known by physicians acting in emergency departments, and taking care of RD patients in the emergency setting can prove challenging for them. Emergency situations can result from the rare disease itself or be unrelated to it, but most often need particular measures to be taken. In order to guide the emergency practitioner in a very practical way to take care of a patient with a diagnosis of a rare disease, a collection of emergency guidelines is being developed by Orphanet. They are produced by French reference centres for rare diseases and reviewed by emergency practitioners. Patient organizations are also involved in their production so as to make sure that relevant non-medical aspects of care are taken into account. Emergency guidelines are organized in two sections dedicated to the pre-hospital setting and to the hospital emergency department respectively. So far more than 50 guidelines are available in French and are being translated and adapted in other European languages. They have been downloaded more than 340,000 times in 2013 from the Orphanet website (http://www.orpha.net). They are also available in the Orphanet mobile application (developed for iOS and Android), in order to allow emergency practitioners to have timely information in the pre-hospital setting.

Besides of emergency guidelines, the French Ministry of Health has produced personal emergency cards for a number of rare diseases during the first national plan for rare diseases. These cards include summarized recommendations in case of emergency intended to physicians, as well as relevant personal information to be completed by a practitioner in the centre of reference. It includes some recommendations for patients intended to prevent emergency situations related to the disease. Emergency cards are also available on the Orphanet website.

